# Progress in Typhoid Fever Epidemiology

**DOI:** 10.1093/cid/ciy846

**Published:** 2019-02-15

**Authors:** John A Crump

**Affiliations:** Centre for International Health, University of Otago, Dunedin, New Zealand

**Keywords:** epidemiology, death, incidence, typhoid fever, *Salmonella*

## Abstract

*Salmonella enterica* subspecies *enterica* serovar Typhi (*Salmonella* Typhi) is the cause of typhoid fever and a human host–restricted organism. Our understanding of the global burden of typhoid fever has improved in recent decades, with both an increase in the number and geographic representation of high-quality typhoid fever incidence studies, and greater sophistication of modeling approaches. The 2017 World Health Organization Strategic Advisory Group of Experts on Immunization recommendation for the introduction of typhoid conjugate vaccines for infants and children aged >6 months in typhoid-endemic countries is likely to require further improvements in our understanding of typhoid burden at the global and national levels. Furthermore, the recognition of the critical and synergistic role of water and sanitation improvements in concert with vaccine introduction emphasize the importance of improving our understanding of the sources, patterns, and modes of transmission of *Salmonella* Typhi in diverse settings.

*Salmonella enterica* subspecies *enterica* serovar Typhi (*Salmonella* Typhi) is the cause of typhoid fever. Together, *Salmonella* Typhi and *Salmonella* serovar Paratyphi A are the major agents of enteric fever. Like other typhoidal *Salmonella* serovars, *Salmonella* Typhi is a human host–restricted organism. The role of water as a vehicle for typhoid fever has been appreciated since the late 1800s [1, 2] and the role of food not long after [3]. Our understanding of the global burden of typhoid fever has improved in recent decades, with an increase in both the number and geographic representation of high-quality typhoid fever incidence studies, and greater sophistication of modeling approaches. The 2017 World Health Organization (WHO) Strategic Advisory Group of Experts on Immunization (SAGE) recommendation for the introduction of typhoid conjugate vaccines (TCVs) for infants and children aged >6 months in typhoid-endemic countries [4] is likely to require further improvements in our understanding of typhoid burden not only at the global level, but also at the national and subnational levels. Furthermore, the recognition of the critical and synergistic role of water and sanitation improvements in concert with vaccine introduction [5] emphasizes the need to improve our understanding of the sources, patterns, and modes of transmission of *Salmonella* Typhi in diverse local settings. This manuscript summarizes current knowledge, areas of progress, and future directions for work in these areas of *Salmonella* Typhi epidemiology.

## CHAIN OF INFECTION

### Reservoir

Humans are the reservoir (defined as the habitat in which the agent normally lives, grows, and multiplies) of *Salmonella* Typhi. *Salmonella* Typhi has limited capacity to multiply outside of the human host, but it may survive for extended periods in the environment [6]. Acute *Salmonella* Typhi infection presents as typhoid fever. Typhoid fever may be difficult to distinguish clinically from other febrile illnesses. If untreated, intestinal, neuropsychiatric, and other complications develop in some patients. However, acute infection can also be mild and self-limited. Human challenge studies demonstrate that fecal shedding and even bacteremia may occur in the absence of clinical signs of typhoid fever [7].

### Portal of Exit, Route of Infection, and Source

Feces represent the major portal of exit of *Salmonella* Typhi, although shedding in urine has also been documented [8]. *Salmonella* Typhi may be shed in the stool or urine during and following both clinical and subclinical acute infection. Shedding may be temporary or chronic. Temporary shedding may be acute or convalescent. A convalescent carrier sheds *Salmonella* Typhi for ≥3–12 months after the onset of acute illness. A chronic carrier sheds typhoid bacilli for >12 months after onset of acute illness. Practically speaking, a chronic carrier may be defined as someone with no history of typhoid fever or someone who had the disease >1 year previously, who has fecal or urine cultures positive for *Salmonella* Typhi separated by at least 48 hours. The relative contribution of temporary shedding versus shedding from chronic carriers to new infections remains an unanswered yet critical question for typhoid control and elimination. Chronic carriers are known to be a major source of domestically acquired *Salmonella* Typhi infections in countries with low typhoid incidence [9]. However, “Typhoid Mary” [3] has assumed a place in both popular and medical consciousness that belies the potentially greater contribution to transmission of temporary shedding in settings of high typhoid incidence. *Salmonella* Typhi transmission is by the fecal–oral route. Water and food contaminated by human feces are the major sources (defined as the places from which the agent is transferred to a host) of *Salmonella* Typhi. The human reservoir is considered to occasionally be the source of *Salmonella* Typhi, and ingestion of human feces during oral–anal sex has been implicated [10].

### Mode and Patterns of Transmission

The mode of *Salmonella* Typhi transmission is considered to be largely indirect and predominantly vehicle-borne through contaminated water or food [11]. Water and food usually serve as passive vehicles for *Salmonella* Typhi. While *Salmonella* Typhi may survive for extended periods on vehicles, multiplication of *Salmonella* Typhi in water and food is uncommon [6]. Some group *Salmonella* Typhi transmission into 2 broad patterns. In short-cycle transmission, food and water are contaminated by fecal shedding in the immediate environment, and transmission is mediated through inadequate hygiene and sanitation measures. In long-cycle transmission there is contamination of the broader environment, such as pollution of untreated water supplies by human feces and use of raw human feces or untreated sewage as a crop fertilizer [12]. Epidemiologic investigations underscore the important role of chronic carriers in short-cycle foodborne typhoid outbreaks in countries with low typhoid incidence [9], and the potentially large scale of long-cycle waterborne transmission in many high-incidence settings [13]. The means by which the source is contaminated and type of vehicles involved vary considerably from location to location, underscoring the importance of local epidemiologic investigations for informing nonvaccine control measures.

### Portal of Entry and Host

The portal of entry for *Salmonella* Typhi infection is the mouth, usually through ingestion of fecally contaminated water or food. Infection occurs in a susceptible human host. The incubation period shortens and the risk for infection and disease increases with the ingested dose [14]. Gastric acid provides an important barrier to *Salmonella* Typhi accessing the small intestinal mucosa and, in turn, the reticuloendothelial system. Natural and vaccine-induced immunity provide partial protection against typhoid fever [15].

## BURDEN OF DISEASE

Burden of disease represents the impact of a health problem, and is often expressed as disability-adjusted life-years (DALYs). DALYS are calculated by adding years of life lost (YLL) from dying early to years lost due to disability (YLD). The YLL is calculated by multiplying the number of deaths due to a condition by the standard life expectancy at the age of death. YLD is determined by multiplying disease incidence, by the disease’s disability weight, and the duration of illness until remission or death [16]. Unlike diarrhea and pneumonia where a syndrome-wide “envelope” of DALYs is established and then attributed to specific causes, there is presently no DALY envelope for febrile illness. Consequently, a “natural history approach” is taken to estimate the burden of febrile illnesses such as typhoid fever. Here, pathogen-specific studies of disease incidence, complications, and deaths are sought to develop estimates of burden [17]. A summary of typhoid fever burden of disease-related studies from 1980 through 2016 is provided in Table 1. 

**Table 1.** Global Estimates of Typhoid Fever Incidence, Disability, and Death, 1980–2016

	Year										
	1980	1996	2000	2010	2010	2010	2010	2013	2015	2015	2016
Origin (reference[s])	WHO [18]	WHO [19]	CDC [35]	IVI [20]	JHU [21]	WHO [22]	IHME [23, 24]	IHME [25–27]	IHME [28–30]	Yale [31]	IHME [32–34]
Coverage	Global, except China	Global	Global	LMIC, unadjusted^a^	Global	Global	Global, including paratyphoid	Global	Global	LMIC	Global
Illnesses, millions (95% UI or CI)	12.5	16.0	21.7	20.6 (17.5–24.2)	26.9 (18.3–35.7)	21.0 (7.8–44.9)	…	11.0 (9.6–14.4)	12.5 (10.9–14.3)	17.8 (6.9–48.4)	11.8 (10.2–13.6)
DALYs, thousands (95% UI or CI)	…	…	…	…	…	10 292 (3805–22 028)	12 239 (1702–23 043)	11 128 (6014–18 315)	10 576 (5896–17 598)	…	8843 (4902–14 436)
Deaths (95% UI or CI)	…	600 000	216 510	223 000 (131 000–344 000)	223 000 (131 000–344 000)	144 890 (53 519–309 903)	190 200 (23 800–359 100)	160 700 (85 900–268 000)	148 800 (81 900–249 700)	…	128 200 (70 100–210 200)

Abbreviations: CDC, US Centers for Disease Control and Prevention; CI, confidence interval or credible interval; DALY, disability-adjusted life-years; IHME, Institute for Health Metrics and Evaluation; IVI, International Vaccine Institute; JHU, Johns Hopkins University; LMIC, low- to middle-income country; UI, uncertainty interval; WHO, World Health Organization.

^a^The authors estimated the number of typhoid fever cases in LMICs in 2010 after adjusting for water-related risk was 11.9 million (95% confidence interval [CI], 9.9–14.7 million) cases with 129 000 (95% CI, 75 000–208 000) deaths.

### Incidence

The incidence of a disease is the number of new cases per population per unit time. For typhoid fever, incidence is usually expressed as cases per 100 000 population per year. Typhoid fever incidence is often classified as low, medium, high [35], and, more recently, very high [31], corresponding to incidence bands of <10, 10–100, >100–<500, and ≥500 per 100 000 per year, respectively. For burden of disease calculations, population-based incidence measured by active disease surveillance accounting for blood culture insensitivity is used [35]. Studies combining sentinel healthcare facility surveillance with healthcare utilization surveys to account for underascertainment are increasingly done to approximate population-based disease incidence with lower resource investment [17, 36].

Early attempts to estimate the global burden of typhoid fever were hampered by the very limited number of contemporary population-based studies of typhoid fever incidence using blood culture confirmation from typhoid-endemic areas [18, 19]. Data from the control arm of the burgeoning number of typhoid vaccine trials were central to the 2000 estimate of global typhoid incidence, but their use was tempered by concern for bias due to the preference for conducting vaccine trials in high-incidence settings [35]. Since that time, a number of studies [37–39] have been completed that bolster data on typhoid fever incidence from an increasingly diverse range of locations. Such studies include those that are truly population-based, with household surveillance for fever and blood culture in the home or by active referral to healthcare facilities [37]. In addition, a number of recent incidence studies rely on healthcare facility–based surveillance supplemented with healthcare utilization surveys that provide multipliers to account for underascertainment related to healthcare access among febrile persons [39].

There is growing recognition of the considerable variation in typhoid fever incidence that may occur in place and over time. Whereas in 2000 typhoid fever appeared to be less common or underascertained in Africa compared with Asia [35, 40], more recent studies confirm that typhoid fever incidence is high in some parts of Africa [37, 39]. Furthermore, typhoid has been demonstrated to occur at high incidence after years of little disease in some locations [41] while declining markedly from high incidence levels elsewhere [39]. Furthermore, *Salmonella* serovars other than Typhi play differing roles by location. Among Asian bloodstream infection studies, both typhoid fever and paratyphoid fever are common [42]. African studies demonstrate that nontyphoidal *Salmonella* invasive disease is often as common or exceeds typhoid fever incidence in some locations [43]. Estimates from the past 5 years indicate that 11.0–17.8 million typhoid fever illnesses occur annually worldwide (Table 1). Notwithstanding substantial improvements and changes in the amount of source data and methods for extrapolation, the annual number of typhoid fever illnesses have not kept pace with global population growth. However, typhoid fever still ranks high among the major causes of infectious disease illness and death [32, 33].

### Drivers of Typhoid Fever Incidence

Perhaps not surprisingly, given predominant modes of *Salmonella* Typhi transmission, early 20th century data from large cities in Europe and North America repeatedly demonstrated a reduction in typhoid fever illnesses and deaths ecologically associated with measures to improve the microbiologic quality of drinking water [2]. It is likely that similar health gains could be achieved in typhoid-endemic countries if human feces could reliably be excluded from drinking water and food. In 1990, the regions of sub-Saharan Africa, South and Southeast Asia, and Oceania had the lowest population coverage with improved water and sanitation facilities. By 2015, water and sanitation coverage had increased markedly in South and Southeast Asia, and less so in sub-Saharan Africa [44]. However, during the same period Oceania made little progress in either category and is now thought to experience the highest incidence of typhoid fever of any global region [31].

### Disability and Death

The case fatality risk of typhoid fever was approximately 10%–30% in the preantimicrobial era [45]. With effective antimicrobials, the case fatality risk is usually <1%. Antimicrobial-resistant *Salmonella* Typhi is a major global health concern [46]. Clinical studies demonstrate the association between antimicrobial resistance and poor patient outcomes [47]. One of the most important modifiable contributors to a poor outcome in typhoid fever is a delay in instituting effective antimicrobial treatment. This delay is more likely to occur where typhoid fever is underrecognized as a cause of febrile illness [48], and where empiric antimicrobial therapy does not correspond with patterns of antimicrobial susceptibility of local *Salmonella* Typhi strains.

Population-based studies of typhoid fever incidence are rarely large enough to accurately estimate the prevalence of complications such as intestinal perforation and deaths. The early detection and enhanced clinical management of typhoid fever inherent and appropriate in such studies modifies the occurrence of complications and deaths. Hospital-based studies are likely to be biased toward more severe disease or those with the ability to access hospital-level healthcare [36]. To date, estimates of the proportion of typhoid fever patients developing complications and dying has been based on expert opinion and on systematic reviews of hospital-based studies that have inherent limitations. Research under way at present is anticipated to shore up estimates of typhoid fever complications and deaths.

### Extrapolation, Uncertainty, and Data Gaps

The sophistication of approaches to extrapolating typhoid fever incidence from locations with robust studies to other geographic areas has increased over time. Early efforts used geographic proximity as the basis of extrapolation [35], an approach that may be extended to account for socioeconomic conditions. Subsequently, water-related risk factors for typhoid fever have been used as a basis for extrapolation [20]. More recently, a range of national or subnational characteristics that are not necessarily on the causal pathway for typhoid fever have been used to inform models for extrapolation [31, 49].

Limited incidence data, variation in the quality of incidence studies, and extrapolation introduce considerable uncertainty into global typhoid fever burden estimates. Uncertainty was not formally addressed in early typhoid fever estimates, but is explored by sensitivity analysis or is represented with uncertainty intervals in contemporary efforts (Table 1).

There remain many data gaps related to typhoid fever burden estimation [50]. Key areas where more data are needed include wider geographic representation of typhoid incidence studies, research on typhoid complications and deaths, and a better understanding of typhoid fever incidence and severity among infants and young children.

### Typhoid Fever Among Infants and Young Children

**Figure 1.**
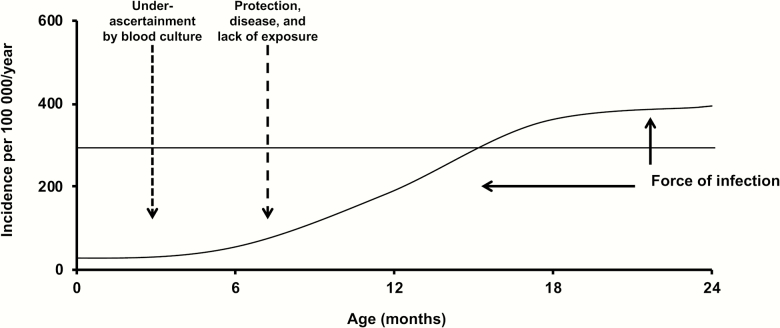
Schematic representation of potential drivers of typhoid fever incidence and uncertainty about typhoid fever incidence among infants and young children <2 years of age.

A recent systematic review of pediatric enteric fever showed that typhoid fever is common among children <5 years of age in many locations [51]. However, the review also highlighted the lack of detailed data on typhoid occurrence in the youngest age groups, especially those <2 years of age. Figure 1 illustrates potential drivers of typhoid fever incidence and uncertainty about typhoid fever incidence among infants and young children <2 years of age. Typhoid fever incidence likely describes a sigmoid curve in this age group, with low incidence in the neonatal period, rising with age. The main drivers of relatively low typhoid incidence among neonates and infants include potential protection from maternal antibodies, the somewhat controversial possibility of less severe disease in younger age groups, and lack of exposure to fecally contaminated water and food vehicles during the period of exclusive or predominant breastfeeding. Force of infection, as measured by population typhoid fever incidence, will have the effect of pushing the peak of disease incidence toward younger age groups and the magnitude of incidence upward [35]. However, typhoid fever may be underascertained in younger age groups through underuse of blood culture in neonates and infants, and inadequate blood volumes for culture and the higher risk for blood culture contamination in the young, when it is attempted (Figure 1).

A growing body of evidence demonstrates a substantial typhoid fever problem among infants and children in settings of high typhoid fever incidence [41]. With TCVs able to protect infants and young children, WHO SAGE recently recommended the use of TCV among those aged >6 months in typhoid-endemic countries [4]. Data on typhoid fever occurrence among infants and young children, as well as other age groups, will be invaluable as countries strive to make evidence-based decisions about whether and at what age to introduce TCVs.

## THE WAY FORWARD

Despite >100 years of research, important gaps remain in our understanding of typhoid fever epidemiology. More data are needed on the contribution of temporary versus chronic carriers to *Salmonella* Typhi transmission, particularly in the low-resource areas where typhoid fever is particularly common. Understanding the proportion of typhoid fever infections that are subclinical and their role, if any, in transmission as well as the efficacy of available typhoid vaccines in preventing shedding is important to designing effective strategies to interrupt transmission. It is apparent that patterns and modes of transmission of *Salmonella* Typhi vary considerably from place to place. Identifying and tracking the relative contributions of short-cycle and long-cycle patterns of transmission, and the relative contributions of water, food vehicles, and other sources to transmission will be central to control strategies in a given location. Presumably reflecting both the remaining uncertainties and the variations in typhoid epidemiology, experts differ in their opinions on the role of different exposure pathways for *Salmonella* Typhi infection by region [52]. At the same time, understanding which components of water, sanitation, and hygiene interventions are most appropriate for preventing typhoid fever in particular locations and globally are highly dependent on such knowledge.

An “envelope” of DALYs for fever without localizing features, modeled on the approach for diarrhea and pneumonia, or other innovations such as the application of serologic surveys to support burden estimation [53] await development and refinement. Consequently, the typhoid fever community must rely on a “natural history” approach to estimating and monitoring the global burden of typhoid fever. Natural history models of typhoid fever are only as good as the quality and representativeness of contributing studies of incidence, complications, and deaths, and the performance of models for data extrapolation. Although the number of incidence studies and the sophistication of modeling have grown, major data gaps remain. In particular, incidence studies are lacking for large geographic areas and among infants and young children. Furthermore, our understanding of the occurrence of complications and deaths and the contribution of antimicrobial resistance to these would benefit from more research. The recent WHO SAGE recommendation for the introduction of TCVs for infants and children aged >6 months in typhoid-endemic countries [4] is likely to increase the demand for such data in many countries seeking to make vaccine decisions. The growing recognition of the considerable variation in the occurrence of typhoid fever in both place [20, 35] and time [41], as well as the anticipated need to measure vaccine impact in countries that introduce TCV, underscores the importance of robust long-term, multiyear typhoid fever surveillance. Ideally, such surveillance should be integrated with surveillance for other major febrile illnesses hitherto overlooked or monitored with independent vertical surveillance programs [48].

## Notes

***Acknowledgments.*** The author thanks Dr Eric D. Mintz, MD, MPH, and Dr Christian S. Marchello, PhD, for comments and suggestions.

***Financial support.*** This work was supported by the Bill & Melinda Gates Foundation (grant numbers OPP1125993 and OPP1158210). J. A. C. is supported by the joint US National Institutes of Health (NIH)/US National Science Foundation Ecology and Evolution of Infectious Disease program (grant number R01 TW009237); the NIH (grant number R01AI121378); the UK Biotechnology and Biological Sciences Research Council (BBSRC) (grant number BB/J010367/1); the UK BBSRC Zoonoses in Emerging Livestock Systems program (award numbers BB/L017679, BB/L018926, and BB/L018845); and the New Zealand Health Research Council through the e-ASIA Joint Research Programme (award number 16/697).

***Supplement sponsorship.*** This supplement was sponsored by Coalition against Typhoid, Sabin Vaccine Institute, Bill & Melinda Gates Foundation.

***Potential conflicts of interest.*** Author certifies no potential conflicts of interest. The author has submitted the ICMJE Form for Disclosure of Potential Conflicts of Interest. Conflicts that the editors consider relevant to the content of the manuscript have been disclosed.
